# Dietary Inflammatory Index and Colorectal Cancer Risk—A Meta-Analysis

**DOI:** 10.3390/nu9091043

**Published:** 2017-09-20

**Authors:** Nitin Shivappa, Justyna Godos, James R. Hébert, Michael D. Wirth, Gabriele Piuri, Attilio F. Speciani, Giuseppe Grosso

**Affiliations:** 1 Cancer Prevention and Control Program, University of South Carolina, Columbia, SC 29208, USA; JHEBERT@mailbox.sc.edu (J.R.H.); wirthm@mailbox.sc.edu (M.D.W.); 2 Department of Epidemiology and Biostatistics, Arnold School of Public Health, University of South Carolina, Columbia, SC 29208, USA; 3 Connecting Health Innovations LLC, Columbia, SC 29201, USA; 4 Inflammation Society, Church Hill, Orpington, London BR6OHH, UK; justynagodos@gmail.com (J.G.); gabriele.piuri@studiospeciani.it (G.P.); attilio.speciani@me.com (A.F.S.); 5 Integrated Cancer Registry of Catania-Messina-Siracusa-Enna, Azienda Ospedaliera Universitaria Policlinico Vittorio Emanuele, 95123 Catania, Italy; giuseppe.grosso@studium.unict.it; 6 NNEdPro Global Centre for Nutrition and Health, St John’s Innovation Centre, Cambridge CB4 0WS, UK

**Keywords:** diet, cytokines, nutrition, inflammation, epidemiology, dietary inflammatory index, colorectal cancer, meta-analysis

## Abstract

Diet and chronic inflammation of the colon have been suggested to be risk factors in the development of colorectal cancer (CRC). The possible link between inflammatory potential of diet, measured through the Dietary Inflammatory Index (DII^®^), and CRC has been investigated in several populations across the world. The aim of this study was to conduct a meta-analysis on studies exploring this association. Data from nine studies were eligible, of which five were case-control and four were cohort studies. Results from meta-analysis showed a positive association between increasing DII scores, indicating a pro-inflammatory diet, and CRC. Individuals in the highest versus the lowest (reference) DII category showed an overall 40% increased risk of CRC with moderate evidence of heterogeneity [relative risk (RR) = 1.40, 95% confidence interval (CI): 1.26, 1.55; *I*^2^ = 69%, *p* < 0.001]. When analyzed as a continuous variable, results showed an increased risk of CRC of 7% for a 1-point increase in the DII score. Results remained unchanged when analyses were restricted to the four prospective studies. Results of our meta-analysis support the importance of adopting a healthier anti-inflammatory diet in preventing CRC. These results further substantiate the utility of DII as tool to characterize the inflammatory potential of diet and to predict CRC.

## 1. Introduction

Colorectal cancer (CRC) is the third most common form of cancer worldwide and is one of the leading causes of cancer-related deaths [1]. Incidence and mortality rates of CRC vary widely with higher incidence rates in developed nations and lower incidence rates in Asia, Africa, and most Latin American countries [2]. Inflammation typically occurs as part of the body’s normal response to tissue insult/injury [3,4]. Chronic inflammation is a persistent condition in which tissue destruction and repair occur simultaneously [5,6], involving continuous recruitment of pro-inflammatory cytokines (associated with increased blood flow to the injured tissue, due to histamine released by damaged mast cells) [3]. Increased levels of these cytokines also are believed to be associated with CRC [7,8,9]. Furthermore, some research suggests a direct association between specific dietary components and inflammation [10,11,12,13]. Various dietary components may be involved in the development of CRC [14]. The 2012 American Institute for Cancer Research/World Cancer Research Fund Continuous Update Project (CUP) reported that consumption of red and processed meat, which are pro-inflammatory, is associated with an increased risk of CRC [14]. Conversely, the consumption of dietary fiber, which is anti-inflammatory, is inversely associated with risk of CRC [14]. Furthermore, other dietary components, such as tea and coffee, which we have found to be anti-inflammatory, have demonstrated various health benefits , including lower cancer incidence [15,16] and mortality [17,18]. Moreover, comprehensive investigations on whole dietary patterns have indicated that unhealthy dietary patterns are associated with higher risk of CRC and adenoma, while healthy diets are associated with lower risk [19,20].

In response to the absence of an instrument that could summarize diets’ ability to influence inflammatory processes, in 2009 researchers at the University of South Carolina developed the first Dietary Inflammatory Index, which was created based on literature published on diet and inflammation through 2007 [21]. In 2014 the new refined and improved Dietary Inflammatory Index (DII^®^) was based on literature published on diet and inflammation through 2010 [22]. The DII categorizes individuals’ diets according to their inflammatory potential on a continuum from maximally pro-inflammatory to maximally anti-inflammatory. A higher DII score indicates a more pro-inflammatory diet, whereas a lower DII score represents a more anti-inflammatory diet. The DII is composed of 45 food parameters, out of which 36 are anti-inflammatory. These include: fiber, alcohol, monounsaturated fatty acids, polyunsaturated fatty acids, omega 3, omega 6, niacin, thiamin, riboflavin, vitamin B6, B12, zinc, magnesium, selenium, vitamin A, vitamin C, vitamin D, vitamin E, folic acid, beta carotene, anthocyanidins, flavan3ols, flavonols, flavanones, flavones, isoflavones, garlic, ginger, onions, thyme, oregano, saffron, turmeric, rosemary, eugenol, caffeine, and tea. The remaining 9 are pro-inflammatory components: energy, carbohydrates, proteins, total fat, trans fat, cholesterol, vitamin B12, saturated fatty acids and iron. As a rule, foods that have low DII scores tend to be flavorful, colorful, nutrient-dense, and calorie-sparse. By contrast, those foods that have high DII scores tend to be flavorless (even though they may have a strong taste, such as sweet), are white or colorless, nutrient-sparse and calorie-dense. The DII was found to predict changes in high sensitivity-C-reactive protein (hs-CRP) in the Seasonal Variation in Blood Cholesterol Study [21,23]. Subsequently, the DII has been used in several studies from around the world to test the effect of diet-associated inflammation on inflammation markers such as CRP, interleukin (IL)-6, and (tumor necrosis factor) and TNF-α-R2 [23,24,25,26,27,28,29,30]. In the Seasonal Variation of Blood Cholesterol Study, higher DII scores were associated with values of hs-CRP > 3 mg/L [odds ratio (OR) = 1.08; 95% confidence interval (CI): 1.01, 1.16, *p* = 0.035 for the 24 hour recall (24 HR) subset; and OR = 1.10; 95% CI: 1.02, 1.19, *p* = 0.015 for the 7-Day Dietary Recall] [23]; in the Women’s Health Initiative, the DII was associated with the four biomarkers with beta estimates comparing the highest with lowest DII quintiles as follows: Interleukin-6: 1.26 (1.15–1.38), *p*_trend_ < 0.0001; tumor necrosis factor alpha receptor 2: 81.43 (19.15–143.71), *p*_trend_ = 0.004; dichotomized hs-CRP (odds ratio for higher vs. lower hs-CRP): 1.30 (0.97–1.67), *p*_trend_ = 0.34; and the combined inflammatory biomarker score: 0.26 (0.12–0.40), *p*_trend_ = 0.0001 [24]. Additionally, the DII has been linked to various health outcomes including cancer incidence [31,32,33]; all-cause, cardiovascular and cancer-specific mortality [34,35,36]; respiratory conditions such as asthma [28,37]; and cognitive disorders [38,39]. The most consistent results have been observed with CRC, with nine studies published exploring this association [40,41,42,43,44,45,46,47,48]. The current meta-analysis aimed to investigate the cumulative association between the inflammatory potential of diet, as estimated by the DII score, and CRC risk based on the results from nine previous studies.

## 2. Methods

### 2.1. Search Strategy and Study Selection

Literature databases including PubMed, SCOPUS, and EMBASE were searched from beginning through July 2017. Relevant keywords related to the DII were searched in combination with keywords related to CRC {[(dietary inflammatory index OR inflammatory diet OR anti-inflammatory diet OR dietary score) AND (colorectal OR colon OR rectal OR rectum)] AND (cancer OR carcinoma OR neoplasm)}. Reference lists of retrieved articles were manually searched by two researchers (G.G. and S.N.). The literature search was limited to English. If more than one article was published using the same cohort, the most recent article with the longest follow-up period was considered. Studies included in this systematic review met all of the following inclusion criteria: (i) focused on humans and had a case-control or a prospective study design; and (ii) evaluated the risk or association between the DII and CRC. The two investigators independently assessed articles for compliance with the inclusion and exclusion criteria and resolved disagreements through consensus.

### 2.2. Data Extraction

The following information was extracted from each study: (i) name of the first author; (ii) year of publication; (iii) study cohort or name; (iv) country; (v) number of participants; (vi) sex of participants; (vii) age range or mean age of the study population at baseline; (viii) follow-up period; (ix) endpoints and cases; (x) measures of risk [hazard ratios (HRs)] or association [odds ratios (ORs)] with 95% confidence intervals (CIs) for the highest versus the lowest category of exposure and for 1-point increase of the DII score (when available); and (xi) covariates used for adjustment.

The quality of observational studies was assessed according to the Newcastle-Ottawa Quality Assessment Scale [49], consisting of three parameters of quality: selection (four points), comparability (two points), and outcome (three points), with a score of seven or more points reflecting high quality.

### 2.3. Statistical Analysis

In this meta-analysis, ORs and HRs were deemed equivalent to relative risks (RRs) [50]. Random- and fixed-effects models were used to calculate pooled RRs with 95% CIs of colorectal cancer for the highest compared to the lowest category of exposure and for a 1-point increase of the DII score. Risk estimates of CRC for 1-point increase of the score (continuous) also were estimated in studies not reporting the measure, but providing sufficient data to estimate it. Heterogeneity was assessed by using the Q test and *I*^2^ statistic. The significance of the Q test was defined as *p* < 0.10. The *I^2^* statistic represents the amount of total variation that could be attributed to heterogeneity. *I^2^* values ≤25%, ≤50%, ≤75% and >75% indicated no, little, moderate, and high heterogeneity, respectively. A sensitivity analysis was conducted by excluding one study at a time in order to assess the stability of results. Subgroup analyses were conducted by tumor localization (colon, rectum), sex, geographical region [North America (*n* = 5), Europe (*n* = 2)], and adjustment for smoking, BMI, physical activity and non-steroidal anti-inflammatory drug (NSAID) use. Publication bias was assessed by visual observation of funnel plots. All analyses were performed with Review Manager (RevMan) version 5.2 (The Nordic Cochrane Centre, The Cochrane Collaboration, Copenhagen, Denmark).

## 3. Results

**Figure 1:**
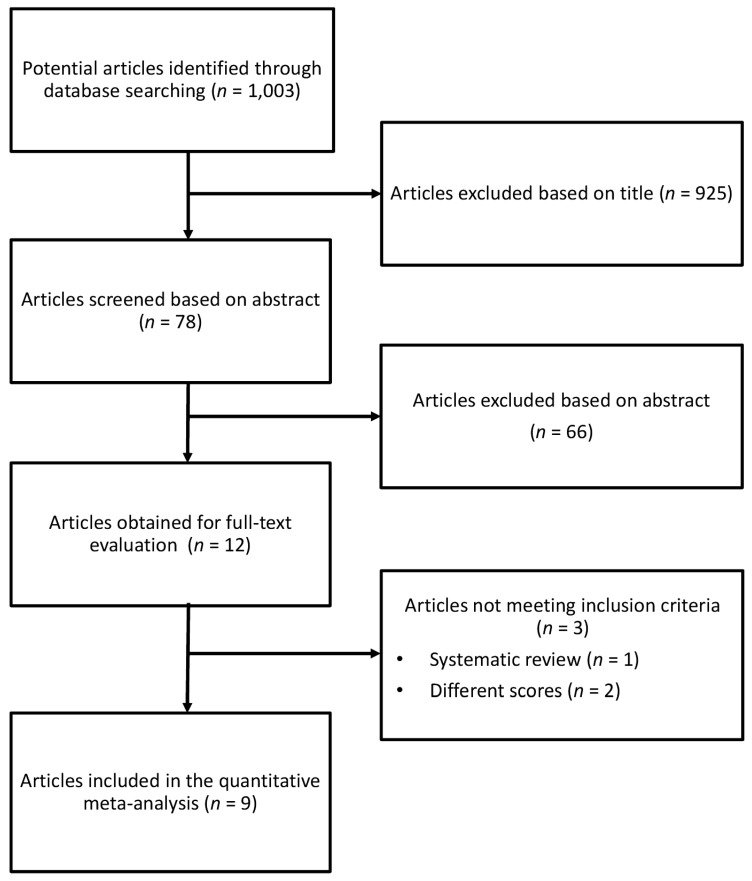
Flow chart and process selection of relevant studies exploring the association between Dietary Inflammatory Index (DII) and risk of colorectal, colon and rectal cancer.

The relevance of studies was assessed with a hierarchical approach on the basis of title, abstract, and the full manuscript. The full process of identification and selection of studies is shown in Figure 1. The search strategy identified 1,003 studies, of which 925 were excluded after review of title, and 66 on the basis of abstract (Figure 1). Of the 12 publications selected, 3 were not included for the following reasons: (1) the article evaluated the association between different dietary score and CRC; (2) the study was a systematic review (and therefore did not present any new finding).

The nine studies selected included a total of 881,612 individuals and 18,888 cases of colorectal cancer available for the present meta-analysis.

Table 1 shows the information extracted from all nine studies included. Five studies had a case-control design [40,44,46,47,48], which comprised 4000 cases and 7288 controls. Four studies were prospective cohorts [41,42,43,45], which comprised 715,088 participants and 14,888 incident cases of colorectal cancer; cohorts included the Iowa Women’s Health Study (IWHS), the Women’s Health Initiative (WHI), The National Institutes of Health–American Association of Retired Persons (NIH-AARP) Diet and Health Study, and the Multiethnic Cohort (MEC). All the studies included covariates that may have significant influence on colorectal cancer, such as age, sex (when not analyzed separately), BMI, education, physical activity, and smoking status. The comprehensive group of covariates used for adjustments are described in Table 1.

**Figure 2:**
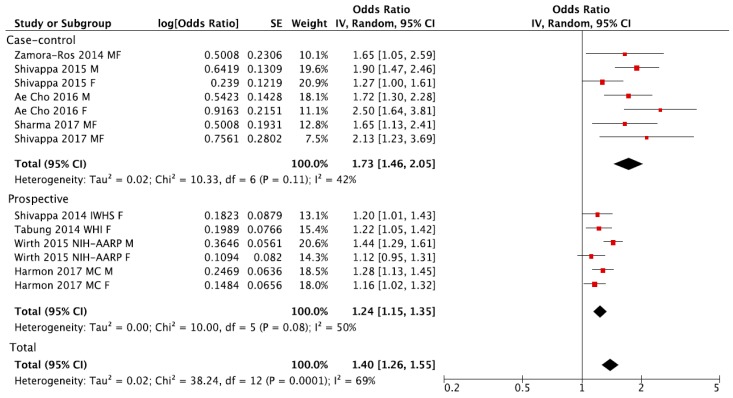
Forest plot of summary relative risks (RRs) of colorectal cancer for the highest versus lowest (reference) category of Dietary Inflammatory Index (DII), for case-control, prospective and all studies.

**Figure 3:**
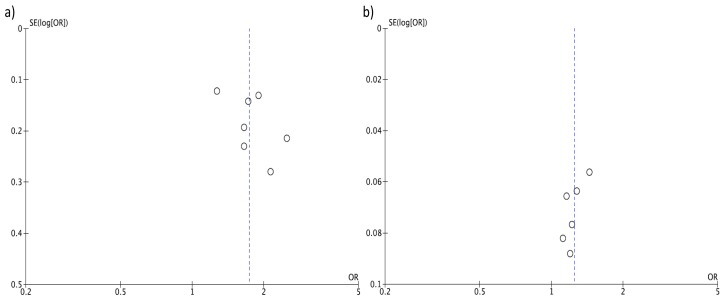

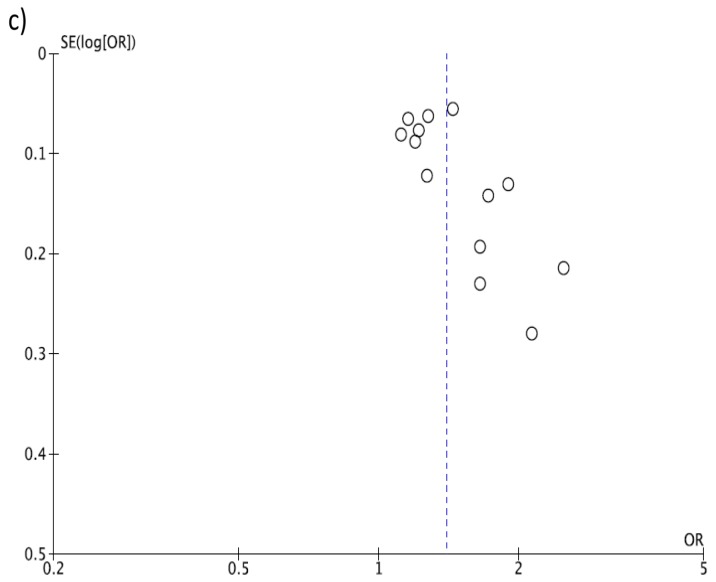
Funnel plots for colorectal cancer risk of the highest versus lowest (reference) category of Dietary Inflammatory Index (DII): (**a**) case-control, (**b**) prospective, and (**c**) all studies.

Individuals in the highest versus the lowest (reference) DII category of exposure had an overall 40% increased risk of colorectal cancer with moderate evidence of heterogeneity (RR = 1.40, 95% CI: 1.26, 1.55; *I*^2^ = 69%, *p* < 0.001; Figure 2). Funnel plot results indicate that case-control studies generally reported higher risk estimates (Figure 3A). Both heterogeneity and funnel plot results (used to evaluate risk of publication bias) were driven by case-control studies (Figure 2 and Figure 3B). However, analysis restricted to prospective cohorts alone showed essentially unchanged risk estimates, with only minor evidence of heterogeneity and no evidence of publication bias (RR = 1.24, 95% CI: 1.15, 1.35; *I*^2^ = 50%, *p* = 0.08; Figure 2 and Figure 3C). When using a fixed-effect model, risk estimates were essentially the same for colorectal cancer (RR = 1.32, 95% CI: 1.26, 1.39; *I*^2^ = 69%, *p* < 0.001), and in separate analysis for prospective cohorts (RR = 1.26, 95% CI: 1.19, 1.33; *I*^2^ = 50%, *p* = 0.08) and case-control studies (RR = 1.68, 95% CI: 1.49, 1.90; *I*^2^ = 42%, *p* = 0.11). Subgroup analyses showed no differences between any of the groups investigated (Table 2).

**Figure 4:**
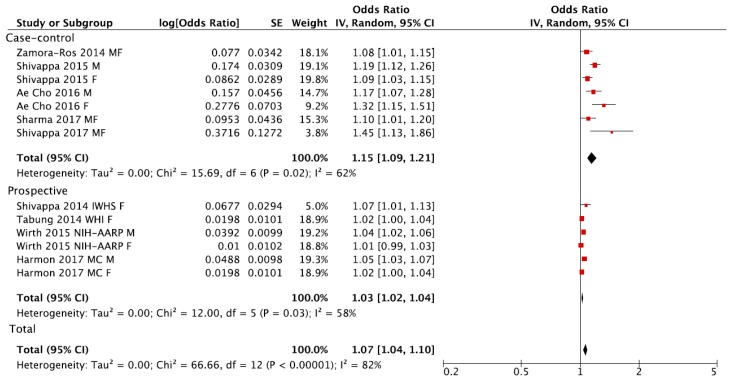
Forest plot of summary relative risks (RRs) of colorectal cancer for a one-point increase of Dietary Inflammatory Index (DII), for case-control, prospective and all studies.

**Figure 5:**
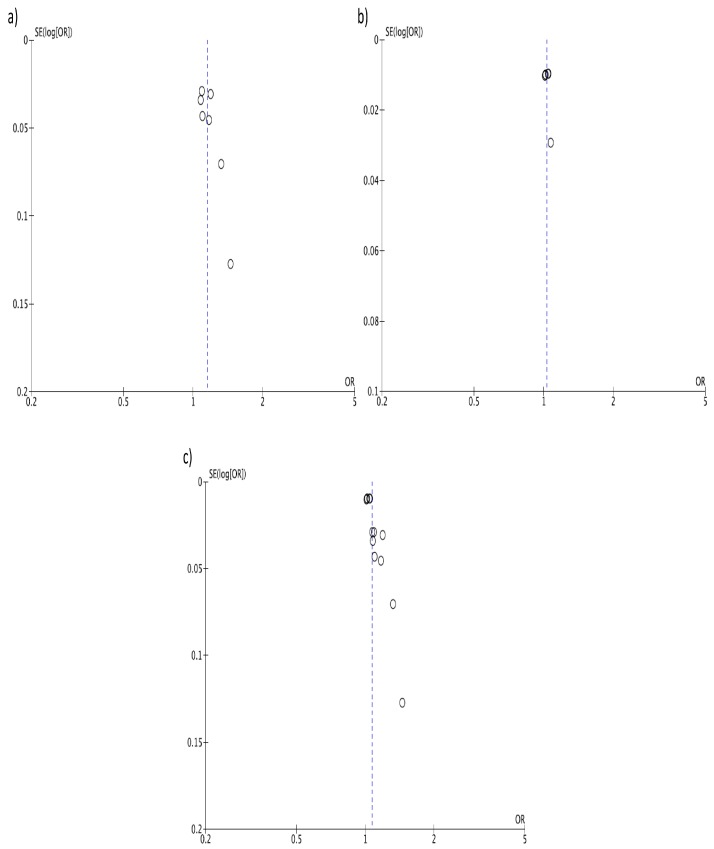
Funnel plots for colorectal cancer risk of a one-point increase of Dietary Inflammatory Index (DII): (**a**) case-control, (**b**) prospective, and (**c**) total studies.

The analysis considering the DII score as a continuous variable showed an increased risk of colorectal cancer of 7% for each 1-point increase of the score, despite the analysis being affected by similar limitations as the previous studies, such as high heterogeneity (Figure 4) and evidence of publication bias based on the funnel plot (Figure 5A). When considering only prospective studies, the association between a 1-point increase of the DII score and risk of colorectal cancer was significant, yet with moderate heterogeneity between results (RR = 1.03, 95% CI: 1.02, 1.04; *I*^2^ = 58%, *p* = 0.03; Figure 4) but no evidence of publication bias at funnel plot (Figure 5C).

**Figure 6:**
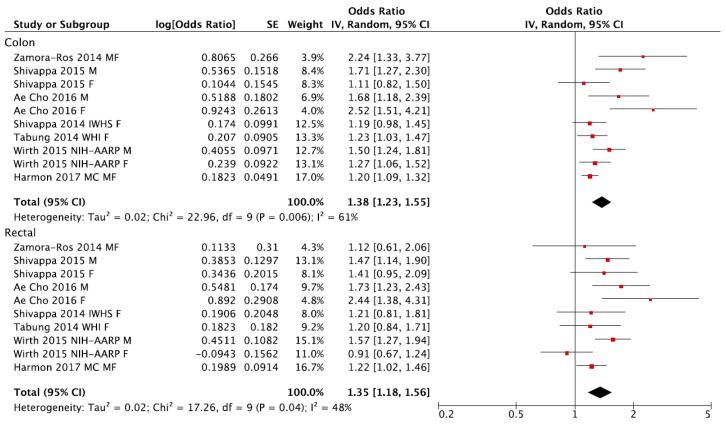
Forest plot of summary relative risks (RRs) of colon and rectal cancer for the highest *versus* lowest (reference) category of Dietary Inflammatory Index (DII).

**Figure 7:**
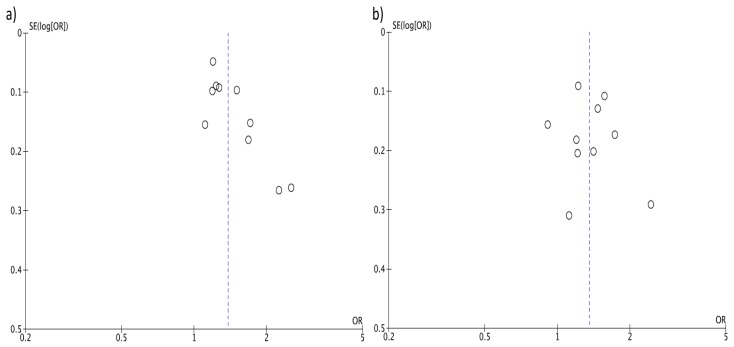
Funnel plots for colon and rectal cancer risk of the highest *versus* lowest (reference) category of Dietary Inflammatory Index (DII): (**a**) colon and (**b**) rectal.

The analyses of separate datasets by tumor location showed similar risk of both colon and rectal cancer for the highest versus the lowest (reference) DII category of exposure, with similar characteristics reported for the general analysis (Figure 6 and Figure 7).

## 4. Discussion

Results from this meta-analysis of nine studies that have examined the association between inflammatory potential of diet, as measured by the DII, and CRC, showed strong evidence of positive association between the DII and CRC. This persisted across tumor location. Therefore, limiting consumption of pro-inflammatory foods, such as red meat, and increasing consumption of anti-inflammatory components, like fruits and vegetables, may play an important role in reducing the risk of CRC. The DII score is calculated from several components and important among them are polyphenols such as flavonoids. Isoflavones, flavanol, flavan-3-ol, anthocyanidins, flavones and flavanones which form the six major groups of flavonoids are included in the DII calculation and all of these are anti-inflammatory and therefore have negative inflammatory effect scores [22].

The DII is a literature-derived population-based dietary index developed specifically to measure the inflammatory potential of individuals’ overall diet across varying populations and dietary assessment methods [30]. There are other dietary indices that exist, such as The Healthy Eating Index (HEI) [51], Alternate Healthy Eating Index (AHEI) [52], Dietary Approaches to Stop Hypertension Score (DASH) [53] and Mediterranean Diet Score (MDS) [54]; and these indices have been examined with CRC as outcome in the past [55,56]. All of these indices represent a dietary scoring pattern that represents healthfulness of the diet. However, none was specifically developed to assess the diet’s inflammatory potential. Another advantage of DII is that it is grounded in the peer-reviewed literature on diet and inflammation and is not dependent on a single study or a few studies within the same or similar populations. Rather, it is based on findings from nearly 2000 articles focusing on laboratory and human studies—from all over the world, employing different study designs and dietary assessment methods. Articles were scored based on the direction of association observed in the article, for example, if in an article garlic significantly reduced levels of CRP then the article would get a score of -1; these articles were then weighted based on the study design. Human studies were given more weight, and clinical trials were assigned the maximum weight of 10. The complete description on the design and development of DII is described in the DII development paper [22]. Findings from the Energy Balance Study indicated that the DII score was negatively correlated with the HEI-2010 (*r* = −0.65, *p* < 0.01), AHEI (*r* = −0.55, *p* < 0.01), and the DASH (*r* = −0.52, *p* < 0.01) [57] and in the Melbourne Collaborative Cohort Study, an inverse correlation was observed between the DII and MDS (*r* = −0.45, *p* < 0.01) [58]. Apart from showing a consistent association between DII and CRC, the DII also was successfully validated with inflammatory markers in several studies across different populations [24,28,29,59,60]. This suggests that the DII represents unique aspects of diet that go beyond what constitutes a generally healthy diet by capturing the specific effect of inflammation compared to other dietary indices. These results provide evidence that the DII is unique in its ability to relate specifically to the core issue of chronic inflammation.

There are several theories to explain the association between the DII and CRC risk; one of the most commonly considered is the effect of pro-inflammatory diet on insulin resistance through increasing systemic inflammation [61]. Another theory suggests the role of diet on local inflammation and oxidation in the colon, which results in focal proliferation and mutagenesis [62]. On the other hand, antioxidant compounds contained in key foods (i.e., fruits, vegetables, coffee, tea, etc.) may exert anti-inflammatory effects, especially locally through the action of local microbiota [63]. Although, we have observed strong evidence of association between DII scores and CRC, two studies examining the association between DII scores and the prevalence or recurrence of colorectal adenoma, which is a precursor of CRC, have produced equivocal results. One, conducted in the in the screening arm of the Prostate, Lung, Colorectal, and Ovarian (PLCO) Cancer Screening Trial, produced positive results, primarily in men [64]. The other, based on data from the Wheat Bran Fiber (WBF) and Ursodeoxycholic Acid (UDCA) Phase III clinical trials, produced null results [65]. Although colorectal adenoma is a risk factor for CRC, most adenomas will not undergo malignant transformation [66,67,68]. More studies are warranted to further understand this association.

Dietary factors can be related to CRC through mechanisms other than inflammation. For example, consumption of red and processed meat increases the risk of CRC through increased levels of the haem iron content [69], N-nitroso compounds formed during the processing of meat [70], of polycyclic aromatic hydrocarbons and heterocyclic aromatic amines from cooking meat at high temperatures [71]. On the other hand, higher fibre intake is believed to be related to a lower risk of colorectal cancer via increase stool bulk, increase stool transit time, and dilute faecal carcinogens [72]. We have looked at the relative effects of the DII score versus other indices such as the healthy eating Index. Usually about 25 to 50% of the variability in one index is explained by the DII [57,73]. So, there clearly are other; that is, not inflammation-related, mechanisms that are operative. It is important to note that the DII would encompass the effect of haem iron, because iron is a pro-inflammatory component of the DII. Factors related to the other effects of fiber are, indeed, related to inflammation. These other mechanisms, along with inflammation, may exacerbate the effect of diet on CRC.

This meta-analysis had some limitations. First, DII score for all the studies was based on self-reports collected from food frequency questionnaires, which are not error-free. We suspect that self-assessments using these instruments in case-control studies are encumbered by recall bias [74], which can lead to a potential misclassification of the exposure. Even in prospective studies such reports may be subject to response set biases [75,76]. Second, DII score was estimated at baseline and diets might change during study follow-up. However, adult dietary habits seem to be relatively stable over time [77]. It has also been shown in the Women’s Health Initiative, where DII was measured at different time points, that changes in DII towards a pro-inflammatory diets are associated with an elevated risk of colon cancer [78]. Third, we observed substantial heterogeneity across studies pooling the CRC risk. The probable reasons for this could be the differences in the number of food parameters considered in the DII score in different studies, demographic characteristics, type of study, and follow-up duration (in the case of prospective studies).

## 5. Conclusions

In conclusion, this meta-analysis suggests that a more pro-inflammatory diet, as estimated by the higher DII score, was independently associated with an increased risk of CRC. Hence, promoting diets low in pro-inflammatory items and rich in anti-inflammatory food components should help in reducing the incidence of CRC. Future research should concentrate on how DII fares in a population with CRC and what effect it would have on CRC-specific mortality.

## Figures and Tables

**Table 1:** Characteristics of studies included in the meta-analysis.

Author, Year	Study Design	Study Cohort, Country	Sex, Age Range/Mean (Years)	No. of Individuals/Controls	No. of Cases	Follow-Up (Years)	No. of Food Parameters to Calculate DII	Adjustments
Shivappa et al. 2014 [45]	Cohort	Iowa Women’s Health Study, USA	Females, 62 ± 4	34,703	1636	19.6	37	Age, BMI, smoking status, pack-years of smoking, education, hormone replacement therapy use, total energy intake, NSAIDs and history of diabetes.
Wirth et al. 2015 [42]	Cohort	NIH-AARP, USA	Both males and females. Age: 62 ± 5.4	489,422	6944	9.1	35	Age, smoking status, BMI, self-reported diabetes, energy intake, physical activity, marital status, education, race and census-based income.
Harmon et al. 2017 [41]	Cohort	Multiethnic Cohort	Both males and females. Age: 45–75	190,963	4388	20	28	Age, sex, BMI, race, self-reported previous diagnosis of diabetes, asthma, and heart attack; use of supplements; smoking status; family history of colon cancer; education; hormone (i.e., estrogen or progesterone) use; aspirin use.
Tabung et al. 2015 [43]	Cohort	Women’s Health Initiative, USA	Females. Age: 50–79	152,536	1920	11.3	32	Age, total energy intake, body mass index, race/ethnicity, physical activity, educational level, smoking status, family history of colorectal cancer, hypertension, diabetes, arthritis, history of colonoscopy, history of occult blood tests, NSAID use, category and duration of estrogen use, category and duration of estrogen & progesterone use, dietary modification trial arm, hormone therapy trial arm and calcium and vitamin trial arm
Shivappa et al. 2015 [48]	Case-control	Italy	Both males and females. Age: Case-60 ± 10 Controls-56 ± 11	4154 controls	1953	-	31	Age, sex, study center, education, BMI, alcohol consumption, physical activity, history of colorectal cancer, and energy intake
Zamora-Ros et al. 2015 [44]	Case-control	Spain	Both males and females. Age: 65.8 ± 12	401 controls	424	-	33	Age, sex, total energy intake, BMI, first-degree family history of CRC, physical activity, tobacco use, and medication use (aspirin and NSAID)
Cho et al. 2016 [46]	Case-control	South Korea	Both males and females. Age: Cases = 56.6 Control = 56.1	1846 controls	923	-	36	Age, sex, BMI, education, family history of colorectal cancer, physical activity, and total energy intake.
Shivappa et al. 2017 [40]	Case-control	Jordan	Both males and females. Age: Cases: 52 ± 11 Controls: 54 ± 12	202 controls	153	-	18	Age, sex, education, physical activity, body mass index, smoking, and family history of colorectal cancer.
Sharma et al. 2017 [47]	Case-control	Canada	Both males and females. Age: Cases: 62 ± 9 Controls: 60 ± 9	685 controls	547	-	29	Age, sex, BMI, physical activity, cholesterol level, triglycerides, family history of CRC, polyps, diabetes, history of colon screening, smoking, alcohol consumption, regular use of NSAIDs, and reported HRT, females only.

**Table 2:** Subgroup analyses of studies reporting risk of colorectal, colon and rectal cancer for the highest *versus* lowest (reference) category of dietary inflammatory index (DII).

Subgroup	No. of Datasets (No. of Studies)	RR (95% CI)	*I*^2^ (%)	*P_heterogeneity_*
Colorectal				
Total	13 (9)	1.40 (1.26, 1.55)	69%	0.0001
Study design				
Prospective	6 (4)	1.24 (1.15, 1.35)	50%	0.08
Case-control	7 (5)	1.73 (1.46, 2.05)	42%	0.11
Gender				
Men	4 (4)	1.51 (1.29, 1.75)	68%	0.02
Women	6 (6)	1.25 (1.10, 1.41)	61%	0.02
Geographical location				
North America	7 (5)	1.26 (1.16, 1.36)	50%	0.06
Europe	3 (2)	1.57 (1.19, 2.07)	61%	0.08
Asia	3 (2)	1.97 (1.57, 2.49)	9%	0.33
Adjustment for smoking				
No	4 (2)	1.74 (1.34, 2.25)	69%	0.02
Yes	9 (7)	1.28 (1.18, 1.40)	52%	0.03
Adjustment for BMI				
No	1 (1)	1.65 (1.13, 2.42)	NA	NA
Yes	12 (8)	1.39 (1.25, 1.54)	70%	0.0001
Adjustment for physical activity				
No	3 (2)	1.22 (1.12, 1.32)	0%	0.55
Yes	10 (7)	1.51 (1.31, 1.74)	70%	0.0004
Adjustment for NSAID				
No	8 (5)	1.50 (1.28, 1.75)	76%	0.0002
Yes	5 (4)	1.25 (1.15, 1.37)	21%	0.28
Colon				
Total	10 (7)	1.38 (1.23, 1.55)	61%	0.006
Study design				
Prospective	5 (4)	1.25 (1.16, 1.35)	11%	0.34
Case-control	5 (3)	1.70 (1.29, 2.24)	62%	0.03
Gender				
Men	3 (3)	1.58 (1.36, 1.83)	0%	0.71
Women	5 (5)	1.27 (1.10, 1.48)	51%	0.09
Geographical location				
North America	5 (4)	1.25 (1.16, 1.35)	11%	0.34
Europe	3 (2)	1.56 (1.06, 2.29)	71%	0.03
Asia	2 (1)	1.97 (1.34, 2.90)	39%	0.20
Adjustment for smoking				
No	4 (2)	1.62 (1.20, 2.19)	66%	0.03
Yes	6 (5)	1.29 (1.16, 1.43)	46%	0.10
Adjustment for BMI				
No	0 (0)	NA	NA	NA
Yes	10 (7)	1.38 (1.23, 1.55)	61%	0.006
Adjustment for physical activity				
No	2 (2)	1.20 (1.10, 1.31)	0%	0.94
Yes	8 (5)	1.48 (1.27, 1.72)	59%	0.02
Adjustment for NSAID				
No	7 (4)	1.43 (1.23, 1.66)	58%	0.03
Yes	3 (3)	1.29 (1.07, 1.56)	62%	0.07
Rectal				
Total	10 (7)	1.35 (1.18, 1.56)	48%	0.04
Study design				
Prospective	5 (4)	1.23 (1.03, 1.47)	54%	0.07
Case-control	5 (3)	1.55 (1.30, 1.85)	7%	0.36
Gender				
Men	3 (3)	1.56 (1.35, 1.81)	0%	0.75
Women	5 (5)	1.28 (0.97, 1.69)	59%	0.05
Geographical location				
North America	5 (4)	1.23 (1.03, 1.47)	54%	0.07
Europe	3 (2)	1.41 (1.15, 1.73)	0%	0.72
Asia	2 (1)	1.90 (1.41, 2.56)	3%	0.31
Adjustment for smoking				
No	4 (2)	1.60 (1.34, 1.91)	4%	0.37
Yes	6 (5)	1.22 (1.04, 1.44)	43%	0.12
Adjustment for BMI				
No	0 (0)	NA	NA	NA
Yes	10 (7)	1.35 (1.18, 1.56)	48%	0.04
Adjustment for physical activity				
No	2 (2)	1.22 (1.03, 1.43)	0%	0.97
Yes	8 (5)	1.40 (1.17, 1.68)	54%	0.03
Adjustment for NSAID				
No	7 (4)	1.43 (1.18, 1.73)	58%	0.03
Yes	3 (3)	1.21 (1.04, 1.41)	0%	0.96

## References

[1] IARC Globocan 2012: Estimated Cancer Incidence, Mortality and Prevalance Worldwide 2012. http://globocan.iarc.fr/Pages/fact_sheets_cancer.aspx.

[2] Vogel V.G., McPherson R.S. (1989). Dietary epidemiology of colon cancer. Hematol. Oncol. Clin. N. Am..

[3] Keibel A., Singh V., Sharma M.C. (2009). Inflammation, microenvironment, and the immune system in cancer progression. Curr. Pharm. Des..

[4] Pan M.H., Lai C.S., Dushenkov S., Ho C.T. (2009). Modulation of inflammatory genes by natural dietary bioactive compounds. J. Agric. Food Chem..

[5] Coussens L.M., Werb Z. (2002). Inflammation and cancer. Nature.

[6] Philip M., Rowley D.A., Schreiber H. (2004). Inflammation as a tumor promoter in cancer induction. Semin. Cancer Biol..

[7] Chung Y.-C., Chang Y.-F. (2003). Serum interleukin-6 levels reflect the disease status of colorectal cancer. J. Surg. Oncol..

[8] Terzic J., Grivennikov S., Karin E., Karin M. (2010). Inflammation and colon cancer. Gastroenterology.

[9] Toriola A.T., Cheng T.Y., Neuhouser M.L., Wener M.H., Zheng Y., Brown E., Miller J.W., Song X., Beresford S.A.A., Gunter M.J. (2013). Biomarkers of inflammation are associated with colorectal cancer risk in women but are not suitable as early detection markers. Int. J. Cancer.

[10] Santos S., Oliveira A., Lopes C. (2013). Systematic review of saturated fatty acids on inflammation and circulating levels of adipokines. Nutr. Res..

[11] Bordoni A., Danesi F., Dardevet D., Dupont D., Fernandez A.S., Gille D., dos Santos C.N., Pinto P., Re R., Rémond D. (2015). Dairy products and inflammation: A review of the clinical evidence. Crit. Rev. Food Sci. Nutr..

[12] Barbaresko J., Koch M., Schulze M.B., Nothlings U. (2013). Dietary pattern analysis and biomarkers of low-grade inflammation: A systematic literature review. Nutr. Rev..

[13] Simopoulos A.P. (2002). The importance of the ratio of omega-6/omega-3 essential fatty acids. Biomed. Pharmacother..

[14] Continuous Update Project Report Food, Nutrition, Physical Activity, and the Prevention of Colorectal Cancer. http://www.aicr.org/assets/docs/pdf/reports/Second_Expert_Report.pdf.

[15] Vuong Q.V. (2014). Epidemiological evidence linking tea consumption to human health: a review. Crit. Rev. food Sci. Nutr..

[16] Grosso G., Godos J., Galvano F., Giovannucci E.L. (2017). Coffee, Caffeine, and Health Outcomes: An Umbrella Review. Annu. Rev. Nutr..

[17] Zhang C., Qin Y.Y., Wei X., Yu F.F., Zhou Y.H., He J. (2015). Tea consumption and risk of cardiovascular outcomes and total mortality: A systematic review and meta-analysis of prospective observational studies. Eur. J. Epidemiol..

[18] Grosso G., Micek A., Godos J., Sciacca S., Pajak A., Martinez-Gonzalez M.A., Giovannucci E.L., Galvano F. (2016). Coffee consumption and risk of all-cause, cardiovascular, and cancer mortality in smokers and non-smokers: A dose-response meta-analysis. Eur. J. Epidemiol..

[19] Grosso G., Bella F., Godos J., Sciacca S., Del Rio D., Ray S., Galvano F., Giovannucci E.L. (2017). Possible role of diet in cancer: Systematic review and multiple meta-analyses of dietary patterns, lifestyle factors, and cancer risk. Nutr. Rev..

[20] Godos J., Bella F., Torrisi A., Sciacca S., Galvano F., Grosso G. (2016). Dietary patterns and risk of colorectal adenoma: A systematic review and meta-analysis of observational studies. J. Hum. Nutr. Diet..

[21] Cavicchia P.P., Steck S.E., Hurley T.G., Hussey J.R., Ma Y., Ockene I.S., Hébert J.R. (2009). A new dietary inflammatory index predicts interval changes in high-sensitivity c-reactive protein. J. Nutr..

[22] Shivappa N., Steck S.E., Hurley T.G., Hussey J.R., Hebert J.R. (2014). Designing and developing a literature-derived, population-based dietary inflammatory index. Public Health Nutr..

[23] Shivappa N., Steck S.E., Hurley T.G., Hussey J.R., Ma Y., Ockene I.S., Tabung F., Hebert J.R. (2014). A population-based dietary inflammatory index predicts levels of C-reactive protein in the Seasonal Variation of Blood Cholesterol Study (SEASONS). Public Health Nutr..

[24] Tabung F.K., Steck S.E., Zhang J., Ma Y., Liese A.D., Agalliu I., Hingle M., Hou L., Hurley T.G., Jiao L. (2015). Construct validation of the dietary inflammatory index among postmenopausal women. Ann. Epidemiol..

[25] Shivappa N., Hebert J.R., Marcos A., Diaz L.E., Gomez S., Nova E., Michels N., Arouca A., Gonzalez-Gross M., Castillo M.J. (2017). Association between dietary inflammatory index and inflammatory markers in the HELENA study. Mol. Nutr. Food Res..

[26] Shivappa N., Wirth M.D., Hurley T.G., Hebert J.R. (2017). Association between the dietary inflammatory index (DII) and telomere length and C-reactive protein from the National Health and Nutrition Examination Survey-1999-2002. Mol. Nutr. Food Res..

[27] Vahid F., Shivappa N., Hekmatdoost A., Hebert J.R., Davoodi S.H., Sadeghi M. (2017). Association between Maternal Dietary Inflammatory Index (DII) and abortion in Iranian women and validation of DII with serum concentration of inflammatory factors: Case-control study. Appl. Physiol. Nutr. Metab..

[28] Wood L.G., Shivappa N., Berthon B.S., Gibson P.G., Hebert J.R. (2015). Dietary inflammatory index is related to asthma risk, lung function and systemic inflammation in asthma. Clin. Exp. Allergy.

[29] Wirth M.D., Shivappa N., Davis L., Hurley T.G., Ortaglia A., Drayton R., Blair S.N., Hébert J.R. (2017). Construct Validation of the Dietary Inflammatory Index among African Americans. J. Nutr. Health Aging.

[30] Wirth M.D., Burch J., Shivappa N., Violanti J.M., Burchfiel C.M., Fekedulegn D., Andrew M.E., Hartley T.A., Miller D.B., Mnatsakanova A. (2014). Association of a dietary inflammatory index with inflammatory indices and metabolic syndrome among police officers. J. Occup. Environ. Med./Am. Coll. Occup. Environ. Med..

[31] Shivappa N., Hebert J.R., Polesel J., Zucchetto A., Crispo A., Montella M., Franceschi S., Rossi M., La Vecchia C., Serraino D. (2016). Inflammatory potential of diet and risk for hepatocellular cancer in a case-control study from Italy. Br. J. Nutr..

[32] Shivappa N., Hebert J.R., Rosato V., Rossi M., Montella M., Serraino D., La Vecchia C. (2016). Dietary inflammatory index and ovarian cancer risk in a large Italian case-control study. Cancer Causes Control.

[33] Shivappa N., Hebert J.R., Rosato V., Serraino D., La Vecchia C. (2016). Inflammatory potential of diet and risk of laryngeal cancer in a case-control study from Italy. Cancer Causes Control.

[34] Graffouillere L., Deschasaux M., Mariotti F., Neufcourt L., Shivappa N., Hebert J.R., Wirth M.D., Latino-Martel P., Hercberg S., Galan P. (2016). Prospective association between the Dietary Inflammatory Index and mortality: Modulation by antioxidant supplementation in the SU.VI.MAX randomized controlled trial. Am. J. Clin. Nutr..

[35] Shivappa N., Blair C.K., Prizment A.E., Jacobs D.R., Steck S.E., Hebert J.R. (2016). Association between inflammatory potential of diet and mortality in the Iowa Women’s Health study. Eur. J. Nutr..

[36] Shivappa N., Steck S.E., Hussey J.R., Ma Y., Hebert J.R. (2015). Inflammatory potential of diet and all-cause, cardiovascular, and cancer mortality in national health and nutrition examination survey iii study. Eur. J. Nutr..

[37] Maisonneuve P., Shivappa N., Hebert J.R., Bellomi M., Rampinelli C., Bertolotti R., Spaggiari L., Palli D., Veronesi G., Gnagnarella P. (2016). Dietary inflammatory index and risk of lung cancer and other respiratory conditions among heavy smokers in the cosmos screening study. Eur. J. Nutr..

[38] Kesse-Guyot E., Assmann K.E., Andreeva V.A., Touvier M., Neufcourt L., Shivappa N., Hebert J.R., Wirth M.D., Hercberg S., Galan P. (2017). Long-term association between the dietary inflammatory index and cognitive functioning: Findings from the su.Vi.Max study. Eur. J. Nutr..

[39] Sanchez-Villegas A., Ruiz-Canela M., de la Fuente-Arrillaga C., Gea A., Shivappa N., Hebert J.R., Martinez-Gonzalez M.A. (2015). Dietary inflammatory index, cardiometabolic conditions and depression in the seguimiento universidad de navarra cohort study. Br. J. Nutr..

[40] Shivappa N., Hebert J.R., Steck S.E., Hofseth L.J., Shehadah I., Bani-Hani K.E., Al-Jaberi T., Al-Nusairr M., Heath D., Tayyem R. (2017). Dietary inflammatory index and odds of colorectal cancer in a case-control study from Jordan. Appl. Physiol. Nutr. Metab..

[41] Harmon B.E., Wirth M.D., Boushey C.J., Wilkens L.R., Draluck E., Shivappa N., Steck S.E., Hofseth L., Haiman C.A., Le Marchand L. (2017). The dietary inflammatory index is associated with colorectal cancer risk in the multiethnic cohort. J. Nutr..

[42] Wirth M.D., Shivappa N., Steck S.E., Hurley T.G., Hebert J.R. (2015). The dietary inflammatory index is associated with colorectal cancer in the national institutes of health-american association of retired persons diet and health study. Br. J. Nutr..

[43] Tabung F.K., Steck S.E., Ma Y., Liese A.D., Zhang J., Caan B., Hou L., Johnson K.C., Mossavar-Rahmani Y., Shivappa N. (2015). The association between dietary inflammatory index and risk of colorectal cancer among postmenopausal women: Results from the women’s health initiative. Cancer Causes Control.

[44] Zamora-Ros R., Shivappa N., Steck S.E., Canzian F., Landi S., Alonso M.H., Hebert J.R., Moreno V. (2015). Dietary inflammatory index and inflammatory gene interactions in relation to colorectal cancer risk in the bellvitge colorectal cancer case-control study. Genes Nutr..

[45] Shivappa N., Prizment A.E., Blair C.K., Jacobs D.R., Steck S.E., Hebert J.R. (2014). Dietary inflammatory index and risk of colorectal cancer in the iowa women’s health study. Cancer Epidemiol. Prev. Biomark..

[46] Cho Y.A., Lee J., Oh J.H., Shin A., Kim J. (2016). Dietary inflammatory index and risk of colorectal cancer: A case-control study in Korea. Nutrients.

[47] Sharma I., Wang P.P., Zhu Y., Woodrow J.R., Mulay S., Parfrey P.S., McLaughlin J.R., Hebert J.R., Shivappa N., Li Y. (2017). Inflammatory diet and risk of colorectal cancer: A population based case-control study in newfoundland, Canada. Nutrition.

[48] Shivappa N., Zucchetto A., Montella M., Serraino D., Steck S.E., La Vecchia C., Hebert J.R. (2015). Inflammatory potential of diet and risk of colorectal cancer: A case-control study from italy. Br. J. Nutr..

[49] Wells GA S.B., O’Connell D., Peterson J., Welch V., Losos M., Tugwell P. (1999). The Newcastle-Ottawa Scale (nos) for Assessing the Quality of Nonrandomised Studies in Meta-Analyses.

[50] Greenland S. (1987). Quantitative methods in the review of epidemiologic literature. Epidemiol. Rev..

[51] Kennedy E.T., Ohls J., Carlson S., Fleming K. (1995). The healthy eating index: Design and applications. J. Am. Diet. Assoc..

[52] McCullough M.L., Feskanich D., Stampfer M.J., Giovannucci E.L., Rimm E.B., Hu F.B., Spiegelman D., Hunter D.J., Colditz G.A., Willett W.C. (2002). Diet quality and major chronic disease risk in men and women: Moving toward improved dietary guidance. Am. J. Clin. Nutr..

[53] Fung T.T., Chiuve S.E., McCullough M.L., Rexrode K.M., Logroscino G., Hu F.B. (2008). Adherence to a dash-style diet and risk of coronary heart disease and stroke in women. Arch. Intern. Med..

[54] Panagiotakos D.B., Pitsavos C., Stefanadis C. (2006). Dietary patterns: A mediterranean diet score and its relation to clinical and biological markers of cardiovascular disease risk. Nutr. Metab. Cardiovasc. Dis..

[55] Park S.Y., Boushey C.J., Wilkens L.R., Haiman C.A., Le Marchand L. (2017). High-quality diets associate with reduced risk of colorectal cancer: Analyses of diet quality indexes in the multiethnic cohort. Gastroenterology.

[56] Vargas A.J., Neuhouser M.L., George S.M., Thomson C.A., Ho G.Y., Rohan T.E., Kato I., Nassir R., Hou L., Manson J.E. (2016). Diet quality and colorectal cancer risk in the women’s health initiative observational study. Am. J. Epidemiol..

[57] Wirth M.D., Hebert J.R., Shivappa N., Hand G.A., Hurley T.G., Drenowatz C., McMahon D., Shook R.P., Blair S.N. (2016). Anti-inflammatory dietary inflammatory index scores are associated with healthier scores on other dietary indices. Nutr. Res..

[58] Hodge A.M., Bassett J.K., Shivappa N., Hebert J.R., English D.R., Giles G.G., Severi G. (2016). Dietary inflammatory index, mediterranean diet score, and lung cancer: A prospective study. Cancer Causes Control.

[59] Ruiz-Canela M., Zazpe I., Shivappa N., Hebert J.R., Sanchez-Tainta A., Corella D., Salas-Salvado J., Fito M., Lamuela-Raventos R.M., Rekondo J. (2015). Dietary inflammatory index and anthropometric measures of obesity in a population sample at high cardiovascular risk from the predimed (prevencion con dieta mediterranea) trial. Br. J. Nutr..

[60] Julia C., Assmann K.E., Shivappa N., Hebert J.R., Wirth M.D., Hercberg S., Touvier M., Kesse-Guyot E. (2017). Long-term associations between inflammatory dietary scores in relation to long-term c-reactive protein status measured 12 years later: Findings from the supplementation en vitamines et mineraux antioxydants (su.Vi.Max) cohort. Br. J. Nutr..

[61] Festa A., D’Agostino R., Howard G., Mykkanen L., Tracy R.P., Haffner S.M. (2000). Chronic subclinical inflammation as part of the insulin resistance syndrome—The insulin resistance atherosclerosis study (IRAS). Circulation.

[62] Bruce W.R., Giacca A., Medline A. (2000). Possible mechanisms relating diet and risk of colon cancer. Cancer Epidem. Biomar..

[63] Grosso G., Godos J., Lamuela-Raventos R., Ray S., Micek A., Pajak A., Sciacca S., D’Orazio N., Rio D.D., Galvano F. (2017). A comprehensive meta-analysis on dietary flavonoid and lignan intake and cancer risk: Level of evidence and limitations. Mol. Nutr. Food Res..

[64] Haslam A., Wagner Robb S., Hebert J.R., Huang H., Wirth M.D., Shivappa N., Ebell M.H. (2017). The association between dietary inflammatory index scores and the prevalence of colorectal adenoma. Public Health Nutr..

[65] Sardo Molmenti C.L., Steck S.E., Thomson C.A., Hibler E.A., Yang J., Shivappa N., Greenlee H., Wirth M.D., Neugut A.I., Jacobs E.T. (2017). Dietary inflammatory index and risk of colorectal adenoma recurrence: A pooled analysis. Nutr. Cancer.

[66] Chen C.D., Yen M.F., Wang W.M., Wong J.M., Chen T.H. (2003). A case-cohort study for the disease natural history of adenoma-carcinoma and de novo carcinoma and surveillance of colon and rectum after polypectomy: Implication for efficacy of colonoscopy. Br. J. Cancer.

[67] Xirasagar S., Li Y.-T., Burch J.B., Daguise V., Hurley T.G., Hebert J.R. (2014). Reducing colorectal cancer incidence and disparities: Performance and outcomes of a screening colonoscopy program in south carolina. Adv. Public Health.

[68] Xirasagar S., Hurley T.G., Sros L., Hebert J.R. (2010). Quality and safety of screening colonoscopies performed by primary care physicians. Med. Care.

[69] Gilsing A.M., Fransen F., de Kok T.M., Goldbohm A.R., Schouten L.J., de Bruine A.P., van Engeland M., van den Brandt P.A., de Goeij A.F., Weijenberg M.P. (2013). Dietary heme iron and the risk of colorectal cancer with specific mutations in KRAS and APC. Carcinogenesis.

[70] Zhu Y., Wang P.P., Zhao J., Green R., Sun Z., Roebothan B., Squires J., Buehler S., Dicks E., Zhao J. (2014). Dietary n-nitroso compounds and risk of colorectal cancer: A case-control study in newfoundland and labrador and ontario, canada. Br. J. Nutr..

[71] Diggs D.L., Huderson A.C., Harris K.L., Myers J.N., Banks L.D., Rekhadevi P.V., Niaz M.S., Ramesh A. (2011). Polycyclic aromatic hydrocarbons and digestive tract cancers: A perspective. J. Environ. Sci. Health Part C.

[72] Young G.P., Hu Y., Le Leu R.K., Nyskohus L. (2005). Dietary fibre and colorectal cancer: A model for environment—Gene interactions. Mol. Nutr. Food Res..

[73] Navarro S.L., Neuhouser M.L., Cheng T.D., Tinker L.F., Shikany J.M., Snetselaar L., Martinez J.A., Kato I., Beresford S.A., Chapkin R.S. (2016). The interaction between dietary fiber and fat and risk of colorectal cancer in the women’s health initiative. Nutrients.

[74] Gittelsohn J., Shankar A.V., Pokhrel R.P., West K.P. (1994). Accuracy of estimating food intake by observation. J. Am. Diet. Assoc..

[75] Hebert J.R., Ebbeling C.B., Matthews C.E., Hurley T.G., Ma Y., Druker S., Clemow L. (2002). Systematic errors in middle-aged women’s estimates of energy intake: Comparing three self-report measures to total energy expenditure from doubly labeled water. Anna. Epidemiol..

[76] Hebert J.R., Ma Y., Clemow L., Ockene I.S., Saperia G., Stanek E.J., Merriam P.A., Ockene J.K. (1997). Gender differences in social desirability and social approval bias in dietary self-report. Am. J. Epidemiol..

[77] Thompson F.E., Metzner H.L., Lamphiear D.E., Hawthorne V.M. (1990). Characteristics of individuals and long term reproducibility of dietary reports: The tecumseh diet methodology study. J. Clin. Epidemiol..

[78] Tabung F.K., Steck S.E., Ma Y., Liese A.D., Zhang J., Lane D.S., Ho G.Y.F., Hou L., Snetselaar L., Ockene J.K. (2017). Changes in the inflammatory potential of diet over time and risk of colorectal cancer in postmenopausal women. Am. J. Epidemiol..

